# An Old Doctor’s Story

**Published:** 1882-01-20

**Authors:** 


### AN OLD DOCTOR'S STORY.

We were a knot of doctors, enjoying ourselves after a meeting of the Hippocratic Medical Society, whose members were accustomed to assemble once a year for purposes of mutual edification and improvement. Dr. Galen Cupps was in the chair. He was our Nestor, our old man eloquent, a living professional legendary budget. On his face you could read "Entertainment" as plainly as you ever saw it painted on an old-fashioned tavern sign; and to it, after a day of weary rambling over the dusty paths of therapeutic lore, we turned as naturally for refreshment as the tired wayfarer halts before the inviting sign-board waving hospitable welcome to the cheer within.

Genial Dr. Cupps! Like Father Grimes, that good old man, "we'll never see him more." No monument marks his last resting place. The guild of undertakers, possessed they a spark of gratitude, would not suffer this to be. As for his surviving professional brethren, few of us, I fear, have money to spend in that way.

"How do young doctors, as a rule, get their first start?" queried a cynical-looking M. D. at the foot of the table, with a nose as sharp as the point of his own lancet—"leaving out, of course, exceptional cases, like that of a man swallowing a fish-bone, or dropping suddenly into a fit, where there's no choice but to seek the nearest aid. What I ask is, how do people come to trust their lives deliberately in inexperienced hands? What's the philosophy of it?"

"Popular ignorance, probably," suggested one.

"Or cheek in the youngster," another hinted.

"It's mostly *luck*, I think," remarked the chair, whereon had converged a number of inquiring looks.

"Come, doctor, give us your experience on the point," was moved and seconded.

"*Mine* was a case of pure luck," said he.

"Won't you tell us about it?" we entreated.

Dr. Cupps was not the man to refuse.

"Young men now-a-days," he began, enter the profession with other advantages than we old fellows had. The clinics and hospitals, now accessible to students, afford the opportunities to learn much by observation which we were left to find out through experiments upon our own patients.

"Though I took my degree after a creditable examination, I doubt if I could then have distinguished, by inspection, between the incipient stages of chicken-pox and measles. Had I been called to treat a simple case of rheumatism, ten to one I should have found a verdict of white-swelling, and passed a sentence of amputation, without stopping to ask the victim what he had to say against it. My first patient was Percy Topham, a young man who had inherited a splendid constitution, as well as fortune, but was fast making way with both. The case, no doubt, would have fallen into Dingo's hands, for he monopolized the practice thereabout, but for his absence on a distant call.

"'Come quick, sir!' urged the messenger; 'Mr. Percy's taking on at an awful rate.'

"Without staying to inquire further I snatched my hat and sallied forth, quite forgetting, in the excitement, the new pill-bags wherein was stored my stock of samples.

"I arrived to find my patient 'taking on' at an awful rate, sure enough.

"Poker in hand, he was laying about him in a manner highly detrimental to the furniture. He was 'killing snakes,' he said. Had it been dogs, hydrophobia might have been my diagnosis but snakes, I knew, meant delirium tremens. And such was the decision of Mr. Topham's own more practiced judgment, for, turning toward me in a lucid moment,

"'I've got 'em, Doc,' he said.

"I did my best to calm him, assured him I should bring him round, placed my fingers on his pulse, and began to count the beats, but, darting from me, he exclaimed:

"'There goes the biggest snake yet; the old serpent of all!' making a slash with the poker that caused me to dodge into the corner.

"Leaving two to watch him, and accompanied by the man who had summoned me, I hurried home to procure such remedies as I should conclude the case required—a point, I must confess, I was very far from clear upon. I had been reared in a very temperate community, had seen little of intoxication or its effects, and my reading had not borne specially in that direction.

"Before reaching my office, however, I decided what course to take. There was no time to consult books, Besides, I was ashamed to do that in the presence of the man who waited to carry back the physic. He would have taken it for a confession of ignorance, and would have lost no time in proclaiming me a dunce.

"Overhauling my supply of drugs, and taking a little from every one, I produced a mixture, some element of which I hoped might prove of service.

"'Give him a teaspoonful of it every half hour,' I said to the man, handing him the bottle, on which I was careful not to put a label.

"Not caring to be present to witness the effect of my maiden prescription, 'Tell Mr. Topham I'll call in the morning,' I said.

"Whatever my patient did, I passed a bad night. Of all cases in which to make my *debut*, why should the malignant fates send the very one most likely to expose me?

"With many misgivings I presented myself next morning at the patient's door. I was glad to see there was no crape on it. Passing the servant who admitted me, I hurried trembling to the sick-room.

"'Good morning, Doc,' cried the invalid, rising from a sumptuous breakfast, wiping his mouth with one hand and extending the other! 'By George, you did bring me through famously! That stuff was mighty nasty, but it did the business. I'm sound as a dollar this morning.'

"Before I could reply a serving-man entered; the same by whom I had sent the medicine.

"'What's the matter, Dick?' said Topham.

"'Boxer's dead, sir.'

"'Dead! the deuce! There's a go! I suppose you forgot to call at Botts's, the farrier's, for that drench yesterday; just like you, when my back is turned.'

"'No, sir!' the man replied; 'I stopped and got it on my way from the doctor's, and gave it according to directions.'

"'Just my luck!' cried Topham, smiting the table. 'You see, Doc, Boxer was my fastest trotter. I counted on winning a mint of money on him at the coming races, and now he's gone and kicked the bucket. Well, peace to his *manes!*" as the poet says. Here, Dick, hand the doctor that medicine bottle from the mantel. He may as well fill it up again. This morning's luck may set me on a fresh spree, and there's no telling how soon I may need another dose.'

"A glance at the bottle, as I took it, made me start. It bore a label, on which I read, 'Simon Botts, Farrier.'

"'Can it be,' I mentally exclaimed, 'that it was Botts' potion that cured the man, and mine that killed the horse!' It was a strong case of circumstantial evidence, at any rate.

"Quietly pocketing the bottle I went my way. If the truth was as I surmised, it never came out. Topham sounded my praises everywhere, and soon the local death list was pretty equally filled with the names of old Dingo's patients and my own."—[Ex.

